# “Si usted es mujer lea esto que le conviene”: la publicidad de medicamentos de patentes dirigidos a las madres en la prensa colombiana, 1903-1945

**DOI:** 10.1590/S0104-59702024000100011

**Published:** 2024-04-15

**Authors:** Ángela Lucía Agudelo-González

**Affiliations:** i Profesora asociada, Universidad del Tolima; doctoranda en Historia y Artes, Universidad de Granada.Ibagué – Tolima – Colombia alagudelog@ut.edu.co; alagudelog@ut.edu.coalagudelog@ut.edu.co

**Keywords:** Motherhood, Advertising, Childcare, Medicine, Maternidad, Publicidad, Puericultura, Medicamentos

## Abstract

Este artículo analiza la publicidad de medicamentos de patentes dirigida a las madres en la prensa colombiana entre 1903 y 1945. Muestra cómo estos anuncios jugaron un papel importante al momento de moldear a la mujer como una población objeto de consumo, estableciendo una maternidad científica por medio de la medicalización. La metodología incluyó el análisis de los avisos publicitarios en los periódicos *El Tiempo, La Prensa, Rigoletto, El Faro* y *Evolución*. Igualmente, dialogó con la historiografía de la temática producida en Colombia y otras latitudes. Concluyó que la maternidad fue un nicho importante para la venta de medicamentos, provocando la conformación de una idea comercial de lo materno.

## Abstract (en)

This article analyses the advertising of patent medicine directed at mothers, which circulated in newspapers between 1903 and 1945. It demonstrates that these advertisings played an important role in shaping women as a consumer demographic, promoting a scientific approach to motherhood intertwined with health challenges. The methodology employed included the analysis of advertisements in the newspapers El Tiempo, La Prensa, Rigoletto, El Faro and Evolución. Additionally, it examines the historiography of the subject in Colombia and other latitudes. The conclusion of this study asserts that motherhood was a significant target for pharmaceutical industries, leading to a commercial concept of motherhood.

En 1944, el médico Gómez Tejera señalaba, en su columna del diario *La Prensa*, el papel que debían tener las madres en el cuidado infantil. Afirmaba que: “Ella, aunque profana en medicina, puede descubrir muchas enfermedades de los pequeños” (Gómez, 30 mar. 1944, p.9). En 1912, una publicidad del periódico *El Tiempo* anunciaban las Píldoras del Dr. Lovett, las cuales actuaban “directamente sobre la madre conservándole las fuerzas e indirectamente sobre el hijo proporcionándole carnes sólidas, buena salud, y rosadas mejillas” (La maternidad, 17 dic. 1912, p.3). Las fuentes primarias citadas muestran cómo los médicos colombianos y la publicidad de medicamentos buscaron generar un campo de acción sobre la maternidad durante la primera mitad del siglo XX, convirtiéndola en el centro de atención.

Es así, como la maternidad y lactancia en Colombia atravesaron por un proceso de medicalización. Se caracterizó por un mayor control médico a través del conocimiento científico que buscó centrarse en la concepción, el embarazo, parto y la crianza. Esto llevó a los médicos a especializarse cada vez más en ginecología-obstetricia, influenciados por los cambios acaecidos en la bacteriología y la fisiología (Gutiérrez, 2013). Así mismo, buscaron impactar en la lactancia por medio de publicaciones en la prensa, al igual que manuales de lactancia y crianza dirigidos no solo a las madres, sino también a las nodrizas, con el propósito de disminuir los índices de mortalidad infantil asociados a las carencias alimentarias (Márquez, Gallo, 2017; Agudelo-González, Chapman-Quevedo, 2021, p.209).

La intención de influir en la maternidad no fue un proceso particular para Colombia; desde finales del siglo XIX en el mundo occidental se empezó a prestar mayor atención a las madres buscando disciplinarlas y formarlas a través de los consejos médicos (Knibiehler, 2001, p.76), consolidándose la idea de ser madre de manera transnacional, replicándose de nación en nación. En este sentido, el papel del médico cobró relevancia científica en el direccionamiento del proceso de maternidad, desde la concepción hasta la crianza, como lo muestran Apple (1995), Quiroz (2020), Rustoyburu (2016), Sibrian (2016), García (2011), Freire (2008, 2009), Martins (2008) y Palomar (2005).

Las columnas médicas estaban acompañadas, en la prensa local y nacional, de una variedad de avisos de medicamentos, que tenían como propósito mejorar la experiencia alrededor del proceso de maternidad. Estos anuncios utilizaron la maternidad, la niñez y las prácticas de crianzas como el gancho publicitario para vender productos para las dolencias y problemas que presentaban las mujeres y sus hijos. Este tipo de preparados, que copó y atiborró el mercado de publicidad, eran los denominados de patentes que se mostraban como parte del avance científico en la farmacología, y, por lo tanto, emergieron como una “herramienta discursiva para consolidar la lucha contra la enfermedad” (Piqué, 2019, p.1341).

Se denominaban medicamentos de patentes debido a que su forma de “preparación seguía una fórmula personal y no las fórmulas publicadas en los Codex más reconocidos (farmacopea americana, británica, francesa o alemana)” (García, 2006, p.159). Estos se caracterizaban por ocultar su contenido y promovían solución a diversos males, divulgándolos, principalmente, en revistas y periódicos. Además, se diferenciaban de otras formas terapéuticas denominadas “éticas” dedicadas a la terapéutica profesional, que vendían preparados estandarizados de la farmacopea norteamericana y comercializados, principalmente, en el cuerpo médico (Greene, Herzberg, 2010, p.794).

Así, el sector de producción de medicamentos de patentes fue fuertemente cuestionado por el cuerpo médico norteamericano desde principios del siglo XX, debido a que estos ocultaban elementos perjudiciales para la salud y no eran realizados bajo los estándares mínimos para el consumo humano (Denham, 2020, p.103). Debido a esto, el Consejo de Farmacia y Química de la Asociación Médica Estadounidense empezó a revisar y emitir juicios, desde principios del siglo XX, sobre la oferta de nuevos medicamentos en el marco de un cambio de paradigma médico que conllevó a una mayor vigilancia de la publicidad de los medicamentos (Marks, 2000).

Para el caso colombiano, autores como Víctor García, Victoria Estrada y Jorge Márquez abordan el proceso de regulación y control de medicamentos, donde los de patentes tenían un papel central (Estrada-Orrego, García-García, Márquez-Valderrama, 2022; García, 2006, 2007, 2008; Márquez, 2006). Sin embargo, la historia de la farmacia se ha concentrado mayoritariamente en el estudio de la industria farmacéutica desde el enfoque del desarrollo industrial e impacto económico (Mendoza-Ruiz, Oliveira, Paranhos, 2022; Obando, 2017; Rivero, 2005). Y, en menor medida, la historia de los específicos (Arango, 2006) y su publicidad (García, Márquez, 2004).

Sobre las estrategias publicitarias del sector de la industria farmacéutica, para el caso de Argentina, Sedran y Carbonetti (2019) muestran cómo el discurso publicitario canalizó las aspiraciones de los sectores medios argentinos ofreciéndoles una variedad de productos terapéuticos. Sanders (2017) realiza un estudio similar para el caso mexicano, pero, haciendo énfasis en cómo los avisos difundían mensajes contradictorios alrededor de las mujeres, jugando un papel fundamental las categorías de género, raza y clase. Lo mismo sucedió en EEUU, donde existe una amplia variedad de investigaciones alrededor de los medicamentos de patentes y sus estrategias publicitarias, destacándose los trabajos de: Thomas, (1982), Cayleff (1992), Tone (1999), Marcellus, (2008), Greene, Herzberg, (2010), Burt, (2012), Zlotucha (2018), Segal (2020), Denham (2020), los que muestran cómo los medicamentos de patentes concentraron su atención en convencer a las mujeres sobre la necesidad de comprar sus preparados utilizando estereotipos para incentivar la venta de productos muchas veces nocivos e inútiles para ellas.

Otro elemento importante para el análisis de este artículo se concentró en la historia de la publicidad de medicamentos que se convirtieron en un referente importante para entender cómo para el período estudiado existió una sinergia entre esta parte de la industria y el sostenimiento de las empresas editoriales (Eguizábal, 1998, p.209-219; Piqué, 2019, p.1339; Cowen, Helfand, 1999, p.180; Beard, 2013; Donohue, 2006; Navon, 2017). Así mismo, la categoría de género jugó un papel importante al momento de configurar la publicidad farmacéutica. A través de ésta, se transmitían representaciones y roles de la mujer, como: “Maternidad, domesticidad, sumisión, ansiedad y dependencia” (Ignaciuk, 2009, p.5). En este sentido, Coronado (2008), Mendoza (2005) y Suárez (2013) han mostrado cómo la publicidad era una herramienta de socialización ideológica y jerárquica que buscaba formar a la mujer como una posible madre. También, la publicidad farmacéutica cumplió el rol de orientar al personal sanitario sobre cuál era la población objeto del medicamento, llevando a que algunas patologías fuesen señaladas como estrictamente femeninas o masculinas, generando un sesgo de diagnóstico (Ignaciuk, 2014, p.5; Papí, Cambronero, Ruiz, 2007, p.103).

Atendiendo a lo apuntado, en el presente artículo analizo los avisos publicitarios de medicamentos de patentes o específicos que eran dirigidos a las madres y que fueron difundidos por la prensa colombiana entre 1903 y 1945 con el propósito de entender cómo se perfiló esta población como objeto de consumo, buscando una maternidad científica a través de la medicación. Para lograrlo, me tracé una ruta metodológica que llevó a la revisión de la prensa de la época, entre los que se encuentran periódicos de amplia difusión como *El Tiempo*, fundado en 1911 por Alfonso Villegas, con el objetivo de que fuera el órgano de difusión del partido Republicano; inicialmente circulaba dentro de los simpatizantes del Partido, aunque, rápidamente en la década de 1910 fue transformándose y convirtiéndose en la fuente de información nacional y en uno de los periódicos más importante del siglo XX (Aponte, 2017, p.4). Otra fuente de las mismas características fue el diario *La Prensa*. Su primera edición fue en 1929, en Barranquilla. Se constituyó con el objetivo de difundir las ideas del conservadorismo. Aunque, con los años fue adquiriendo un amplio alcance en la región Caribe colombiano, logrando posicionarse como el medio de difusión predilecto por los habitantes de la región (Álvarez, Colpas, González, 2000, p.20).

El resto de la muestra documental está compuesta por *Rigoletto*, fundado en 1902, por Eduardo Ortega y Julio H. Palacio. Era una empresa editorial de carácter familiar y tenía una función cultural (Álvarez, Colpas, González, 2000, p.18). En seguida está *El Faro*, que nació en Ibagué, en 1933, por iniciativa de Eugenio López. Era un periódico mensual que, en su prospecto, declaraba ser independiente, con un alcance limitado, difundiendo noticias locales de carácter político, económico y comercial. Por último, tenemos *Evolución*, fundado en la ciudad de Honda, en 1936, por Filiberto Poveda, con el objetivo de promover y fortalecer los procesos educativos de la población (Pérez, 2007).

En los periódicos escogidos identifiqué la publicidad farmacéutica dirigida a las madres, de los que seleccioné los avisos que aparecieron por primera vez anunciando los productos (46 en *El Tiempo*, 54 en *La Prensa*, 12 en *Rigoletto*, 4 en el *Faro* y 2 en *Evolución*, un total de 118). Con la selección de estos periódicos busqué analizar la publicación de los avisos publicitarios en periódicos de distinto alcance territorial, diferente espectro ideológico y diversidad de públicos. El objetivo es generar una muestra que permita entender las estrategias de marketing utilizadas para dirigirse a las madres colombianas en la venta de medicamentos de patentes durante el periodo estudiado.

En el artículo tuve en cuenta, principalmente, los escritos que acompañaban la presentación del medicamento, los que explicaban en detalle las ventajas del producto. Esto, debido a que la publicidad del periodo se presentaba muchas veces como aviso clasificado debido a que se estaba explorando el uso de la imagen como elemento central de las comunicaciones (Arango, 2007, p.115). Así, cuando la imagen ya era usada como parte de la publicidad, pasaba a emplearse como parte del impacto visual, pero de manera subordinada al texto, el cual debía de ser fácilmente recordable como gancho para captar posibles clientes (Eguizábal, 1998, p.294). Por último, es necesario aclarar que el análisis de este material se realizó teniendo en cuenta la metodología de la investigación histórica con perspectiva de género (Ortiz, 2002, 2006).

El artículo está dividido en dos partes: la primera, observa los medicamentos que fueron dirigidos a las mujeres para desarrollar y acompañar su rol de madre, en esta incluyo fármacos para lograr el embarazo o para poder llevar con tranquilidad la maternidad; la segunda, explora los medicamentos publicitados para los infantes, tomando a la madre como interlocutora, estos se presentaban como productos que ayudaban a la conservación de la salud de sus hijas e hijos.

## Medicamentos para la infertilidad, embarazo y la maternidad

La medicalización de la maternidad se desarrolló en Colombia en medio de un proceso de modernización que inició con un siglo XX atravesado por las secuelas generadas por la Guerra de los Mil Días, que impactó no solo en lo económico y político, sino que generó serios problemas en la salubridad pública nacional. Esto generó que desde el Estado se implementaran mecanismos para atender las condiciones de sanidad del país como lo fueron las comisiones sanitarias, las cuales tenían entre sus objetivos la salud materno-infantil (Tobón, Orrego, 2022). Complementado con las acciones privadas desde principio del siglo XX, en el marco de la intervención de la Rockefeller Foundation, que tuvo un papel importante en el proceso de control de enfermedades infectocontagiosas y la modernización del aparato sanitario colombiano (Téllez, Quevedo, 2022).

Así mismo, entre 1903 y 1945 el país empezó a tener un proceso de crecimiento que lo llevó a vincularse cada vez más con las dinámicas del comercio internacional a través de la venta de café. Con las divisas generadas en este período se pudieron importar bienes de consumo, entre los cuales se encontraba el “comercio de medicamentos profuso y libre, casi todos importados y de composición desconocida” (Estrada-Orrego, García-García, Márquez-Valderrama, 2022, p.94).

En medio de esto, en la prensa colombiana se empezaron a publicar una serie de anuncios dirigidos a las mujeres cuyo eje giraba alrededor de la idea de una maternidad científica, en donde estas eran las encargadas de la higiene y la salud familiar (Apple, 1995; Agostoni, 2005; Cayleff, 1992). Este mercado de procedencia internacional empezó a ser compartido a partir de la década de 1930 con la producción de multivitamínicos colombianos (Obando, 2017; Rivero, 2005). La procedencia extranjera de la mayoría de los fármacos permitió que estos productos se posicionaran en el mercado colombiano debido a que “vendían mejor si su marca de origen era norteamericana, francesa, alemana o inglesa” (García, Márquez, 2004, p.115).

Pero, del extranjero no solo llegaban los productos, también, se importaban las estrategias publicitarias, fenómeno que se presentó en países como México y Argentina, extrapolándose, como veremos a continuación, sin ninguna adaptación al contexto cultural y social de los países receptores, cumpliendo únicamente con la traducción de los anuncios (Sanders, 2017; Sedran, Carbonetti, 2019). Sin embargo, en ocasiones no se realizaba la respectiva traducción, verbigracia de lo apuntado fue la publicidad impresa en inglés, dirigida a la mujer, titulada “To Ladies”, en el periódico *Rigoletto* (To ladies, 9 dic. 1904, p.4).^1^ Estas reproducciones publicitarias de un país a otro eran posibles debido a la idea de una maternidad de carácter transnacional, que permitió a las mujeres sentirte identificadas con el producto sin importar en que parte del mundo se encontraban (Sanders, 2017, p.5) y generar procesos de afinidad con otras madres del extranjero como referentes de una mejor salud.

Es así como entre 1903 y 1930 los anuncios que se publicaban en la prensa estaban dirigidos principalmente a vender la idea de que toda mujer era una potencial madre; el propósito era aumentar la población demandante de los productos ofrecidos. Se utilizaban estrategias publicitarias vigentes a inicio del siglo XX como variar el tamaño de la letra, usar mayúsculas y negritas (Eguizábal, 1998, p.180). Ejemplo de lo apuntado es el aviso de “supositorios Mitchella Vaginales”, de 1919, el cual se titulaba “Si es usted mujer lea esto que le conviene” ^(^Si es usted…, 3 jul. 1919, p.6).^2^ En este se advertía como la infertilidad era uno de los mayores temores que podía enfrentar una mujer, enumerando luego una serie de síntomas como el flujo mucoso, dolor de espalda, cefalea, mirada lánguida, cansancio, nerviosismo, palidez, seguidos de la frase “esta debilidad es la causa de muchos casos de infertilidad” (p.6).

De manera análoga, otra publicidad de 1929 presentaba la infertilidad como causa de desdicha en la mujer y al medicamento como la solución. El aviso preguntaba: “¿Habrá goce mayor que la maternidad?”, y contestaba afirmando: “El don de la fecundidad, poder criar hijos robustos y vigorosos, es pasión de toda mujer” (Habrá goce…, 27 ene. 1929, p.13). La publicidad reducía el rol femenino al ejercicio de la maternidad, utilizando una concepción de que toda mujer quería ser madre, y cuando la mujer no lograba embarazarse utilizaban las emociones para que ella acudiera a los específicos y, así, conseguir la anhelada gestación.

Acompañando la emocionalidad y el imperativo de adquirir los medicamentos, también fue común la reproducción de testimonios en la parte final del anuncio con el objetivo de ratificar la efectividad del producto y ganar credibilidad. Estas certificaciones eran hechas por mujeres extranjeras que suministraban sus datos con el ánimo de que cualquiera pudiera, en teoría, corroborar lo afirmado. Un ejemplo es el caso de: “La señora Fella L. Borja, calle López n. 570 de la ciudad de Santiago de Chile, dice que hacía muchos años no había podido lograr criar ninguna criatura y después de haber tomado dos pomos de ‘Compuesto Mitchella’ tiene una robusta y sana” (El embarazo…, 2 ene. 1917, p.3; destacados en el original).

La reproducción de certificaciones también era de usual empleo en la venta de medicamentos de patentes en general (Arango, 2006; García, Márquez, 2004). Sin embargo, como lo ha mostrado Denham (2020, p.118-119), para EEUU, la reproducción de testimonios en la publicidad dirigida a las mujeres era una estrategia frecuente que permitía que estas pudieran crear una cultura de fidelidad entre compradoras.

Otra gama de medicamentos dirigidos a las mujeres era los que se promocionaban para poder llevar los síntomas del embarazo, como vómitos, fatigas, mal humor, prometiendo eliminar toda molestia como aparecían en los anuncios de Elixir digestivo de Pepsina de Grimault (Elixir digestivo…, 7 nov. 1903), Tónico Carduí (Goza Ud…, 3 sept. 1929, p.2) y el ya mencionado Compuesto Mitchella (Madres de mañana, 5 jul. 1919, p.3). De igual manera, garantizaban que la ingesta del medicamento traería al mundo “criaturas robustas” (Vino y…, 2 sept. 1903, p.1) eslogan que también fue utilizado varios años después por el Compuesto Mitchella en su publicidad de 1917 (El embarazo…, 2 ene. 1917, p.3). Este tipo de ofrecimientos era un gancho publicitario para que las embarazadas se interesaran en el producto y lo consumieran.

Durante la década de 1910 empezó aparecer en la prensa medicamentos que ayudaban a evitar los dolores del parto. Como lo fueron el Compuesto Mitchella, anunciado también para favorecer la gestación, fue publicitado para evitar las molestias de las contracciones (El compuesto…, 10 ene. 1917, p.3). Pero, no era el único, los específicos denominados Tocanalgine-Eutocine (Parto sin…, 18 nov. 1915, p.1) y el Específico del Indio preparado por José M. Fuentes también se anunciaban para aliviar los dolores del parto (Especifico indio…, 11 dic. 1915, p.1).

Dos de estos medicamentos, emplearon estrategias publicitarias recurriendo a la autoridad de un médico extranjero para validar la eficacia del producto. En el caso de Mitchella, utilizaba al Dr. J.H. Dye, el cual, según publicidad, había “demostrado científicamente que ninguna mujer [debía] tener más los dolores del ‘parto’” (El compuesto Mitchella…, 10 ene. 1917, p.3; destacados en el original). Por otra parte, Tocanalgine afirmaba que era preparado por el doctor Pierre Lauret y, además, fue ratificado “por la Academia de Medicina de París en virtud de comunicaciones hechas en su sesión del 21 de junio de 1914 por los profesores M. Ribemont-Dessaignes y M. Ad. Pinard” (Parto sin…, 18 nov. 1915, p.1).

Recurrir a la ciencia, especialmente a la voz de los médicos, les permitió a estos medicamentos vincularse al discurso de la modernidad (Henderson, 2006, p.30-31). Ahora, no es posible discernir qué clase de fórmula fue usada para la preparación de estos productos que evitaban que las mujeres sintieran uno de los dolores más temidos, ya que al ser de patente no estaban obligados a revelarla, por secreto comercial, para proteger su invención y sortear la competencia. Sin embargo, al consultar otras fuentes de la época existen evidencias que la tocanalgina y la antalgina eran derivados de la morfina (Índice, 1915, p.570), que empleados como parte de la analgesia obstétrica llevaba a cuadros de apnea en los recién nacidos (De Eleizegui, 1914, p.3). Los anuncios de estos fármacos no vuelven a encontrarse y concentraron su aparición mientras la Academia Francesa de Medicina discutía su utilidad como analgésico para el parto.

La venta de medicamentos no solo estaba dirigida al embarazo y el parto, la siguiente gama de anuncios fue destinada al postparto. La publicidad afirmaba que después de parir las fuerzas disminuían y sumado al cuidado del infante, era una carga pesada para la mujer. Por eso, recomendaban las píldoras del Dr. Lovett, que eran “el tónico ideal para las madres debilitadas, poniéndolas en situación de asimilar los alimentos que tan necesarios le son, entonando su organismo y permitiéndole cumplir sus maternales obligaciones” (La maternidad, 17 dic. 1912, p.3).

Este medicamento también era recomendado a la mujer que estaba lactando, con el objeto de producir más leche y proveerle vitalidad a su hijo o hija. En el anuncio se afirmaba que una madre saludable impactaba en la salud de sus descendientes (La maternidad, 4 abr. 1913, p.3). En 1919, anunciaban Lactagol bajo la misma premisa de las Píldoras del Dr. Lovett, afirmando que ayudaba a aumentar y mejorar “la leche de las madres y las nodrizas, Asegura[ando] el buen desarrollo de los niños” (Lactagol, 31 ago. 1919, p.7).

Otra estrategia de mercado empleada por las píldoras rosadas del Dr. Williams era usar la recomendación del medicamento entre amigas. En un anuncio publicado en 1912 titulado “Confidencias entre señoras”, se señalaba lo difícil que era “soportar las responsabilidades del matrimonio y la maternidad” (Confidencias…, 9 oct. 1912, p.3) y recomendaban las pastillas para que no “se gaste por completo la salud y el atractivo físico que toda mujer debe conservar” (p.3).

**Figura 1:**
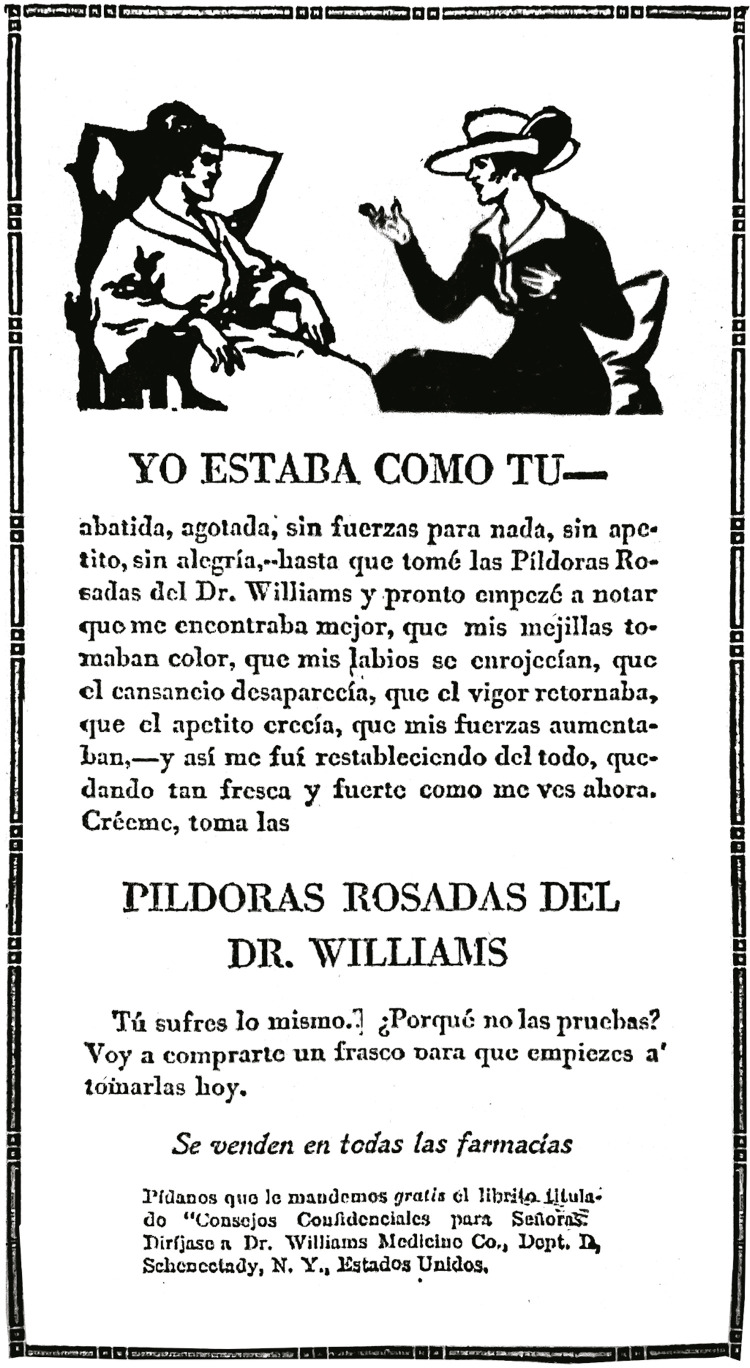
: Yo estaba como tú (Yo estaba…, 26 ago. 1919, p.4)

De igual manera, otro anuncio publicado siete años después (ver Figura 1) apelaba a la misma estrategia como vehículo para la venta de específicos, incentivando a la mujer a automedicarse bajo la premisa de: “Tú sufres lo mismo… ¿Por qué no las pruebas? Voy a comprarte un frasco para que empieces a tomarlas hoy” (Yo estaba…, 26 ago. 1919, p.4). En la imagen que acompañaba el anuncio estaban dos mujeres charlando, una con aspecto de enferma y sentada escuchando atentamente la recomendación de su amiga, la cual viste elegantemente. Con el propósito de captar el mercado femenino intentando reemplazar las fórmulas tradicionales con medicamentos de patentes, este tipo de publicidad utilizó la premisa del autocuidado, que han tenido las mujeres por tradición, donde la cultura oral de los remedios caseros era un común denominador para afrontar los malestares de su familia (Cayleff, 1992, p.304).

Así, los estereotipos de género se convirtieron en recursos publicitarios que se emplearon para lograr atraer la atención de las mujeres, las cuales debían lucir bellas, felices y poder disfrutar su maternidad sin dolores de cabeza, sin que se le quebraran los nervios, llenas de vitalidad. Debido a esto, los medicamentos les permitieron mantener “la frescura de sus mejillas, la elasticidad de su paso, el timbre de su voz” (Una mujer…, 2 sept. 1903, p.6). Dentro de la publicidad se fue configurando entonces un discurso sobre lo que se imaginaba el deber ser de la madre (Davis et al., 2019, p.4) que iba alimentado por las representaciones sociales de la nación colombiana que observó en estos avisos una forma de identificación de un ideal que sería alcanzado, en este caso, mediante la medicación.

Todos los anuncios que iban dirigidos a la madre estaban acompañados de un listado de los síntomas que el medicamento iba a curar. Si bien esta no era una estrategia únicamente de los avisos dirigidos a ellas (García, Márquez, 2004, p.113), se puede identificar que existió una semiología médica dirigida a las mujeres sin importar el específico, estas eran debilidad, nerviosismo, fatiga, anemia, palidez, menstruaciones irregulares, mal humor y cansancio. Por eso, las farmacéuticas fueron configurando enfermedades consideradas propias de la mujer y que dieron forma a los sesgos de diagnóstico y con ello a los roles de género vigentes en la época. Este listado de síntomas permitió a la mujer sentirse identificada y automedicarse, estrategia publicitaria que fue implementada por los comercializadores de medicamentos de patentes vendiendo la idea de una mujer débil necesitada de los productos (Thomas, 1982).

**Figura 2:**
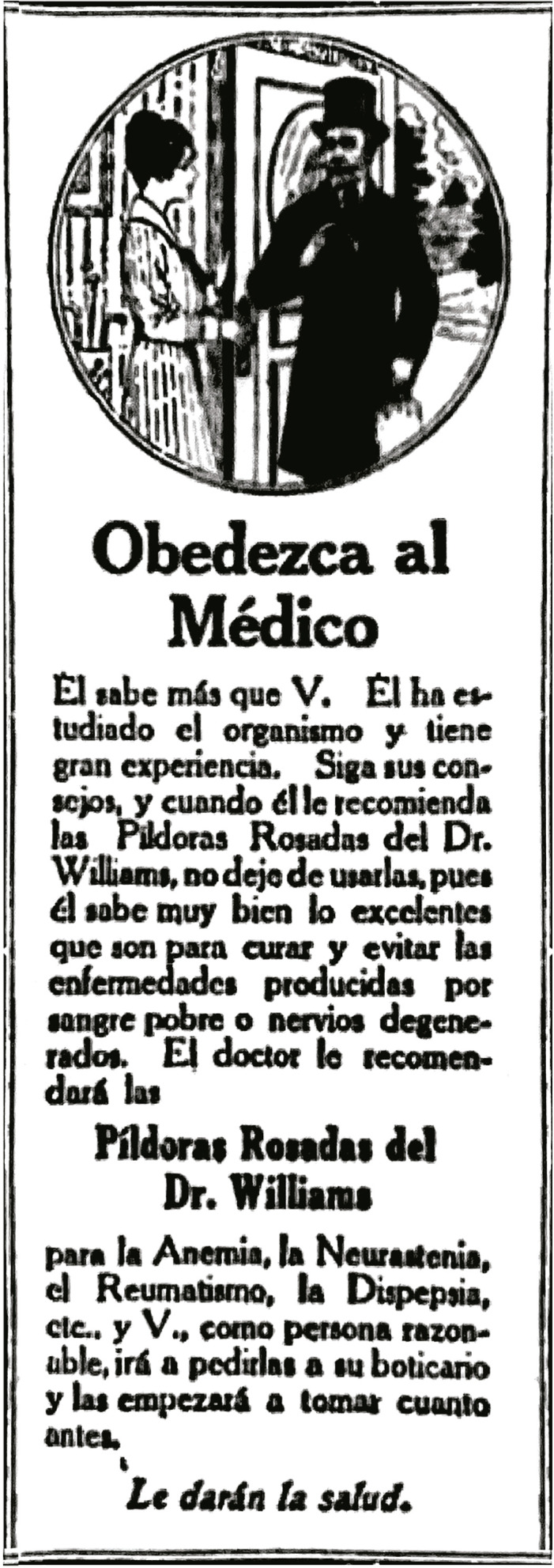
: Obedezca al médico (Obedezca…, 6 jun. 1920, p.7)

También los anuncios tomaban las voces de la autoridad médica de manera genérica, sin hacer referencia a alguien en particular, como sucedió en el anuncio de 1920 (ver Figura 2) que titulaba a modo de orden: “Obedezca al médico” (Obedezca…, 6 jun. 1920, p.7). La publicidad posee una imagen de un hombre con sombrero, gafas y maletín que hacía el papel del médico que hablaba en la puerta de una casa con una mujer joven. En esta publicidad se señalaba la experiencia y los estudios de este para reforzar la recomendación del fármaco. Otras veces utilizaban frases como “considerados por especialistas en enfermedades de mujeres” (Si es usted…, 3 jul. 1919, p.6), o “la ciencia moderna proporciona el remedio que mayor éxito ha dado para tal condición” (Una mujer…, 2 sept. 1903, p.6). Todo esto buscaba validar a través del conocimiento científico la venta de medicamentos producidos para la mujer, usando la figura masculina como símbolo de autoridad clínica y científica en un momento que la profesión médica se consolidaba dentro de la sociedad colombiana (Obregón, 2002).

Ahora bien, la publicidad empleaba estrategias que a primera vista podían ser contradictorias, como se puede leer en las Figuras 1 y 2 y mucho más si se tiene en cuenta que se valieron para vender el mismo medicamento. Una de estas era acudir a la comunidad femenina a través del autocuidado, y la otra utilizar el conocimiento médico como soporte científico de la venta de fármacos. Sin embargo, en un mundo cambiante, este enfoque se convirtió en medidas complementarias, porque apelaban a las ideas de modernidad, progreso y maternidad científica que aspiraban a las capas medias y altas de la sociedad colombiana. Es decir, dentro de la publicidad se acudió a los discursos científicos que junto con los estereotipos de género configuraron un ideal materno que solo podía ser cumplido por mujeres dedicadas a su hogar y al cuidado de su familia y que supieran leer y escribir. Este escenario no es único para el caso colombiano, en otras partes de Latinoamérica se repite la misma situación, como lo muestran las investigaciones de Sanders (2017) y Sedran y Carbonetti (2019).

Es por esto que, entre 1903 y 1930, la publicidad dirigida a la madre varió muy poco, colocando énfasis en una gama de productos que, en teoría, solucionaban las diferentes etapas de la maternidad. Esta oferta terapéutica estaba compuesta de jarabes, supositorios, compuestos, tónicos, píldoras y elixires. Las estrategias publicitarias para vender estos productos se basaban en apelar a la emocionalidad, a los testimonios de otras mujeres y al prestigio médico para construir un modelo ideal femenino alrededor de lo materno. Este modelo funcionó tan bien en este período que el aviso “Preparación de Wampole” titulado “una mujer contenta” fue publicado sin variar una coma en el periódico *Rigoletto* en 1903 (Una mujer…, 2 sept. 1903, p.6) y en el periódico *El Tiempo* en 1929 (Una mujer…, 4 sept. 1929, p.4).

Este modelo publicitario comenzó a variar lentamente debido a la intervención del cuerpo médico que desde finales de la década de 1920 comenzó a presionar al gobierno nacional para lograr una regulación en el mercado de medicamentos de patentes que consistió en una serie de publicaciones, análisis de sustancias en laboratorios y la reglamentación del ejercicio de la farmacia (Estrada-Orrego, García-García, Márquez-Valderrama, 2022, p.97; Téllez, Quevedo, 2022). Inspirados en el proceso desarrollado en EEUU (Denham, 2020, p.123), estos alertaron sobre los peligros para la salud de los colombianos de medicamentos cuestionados por el uso de ingredientes como el alcohol y las drogas heroicas.

Estas medidas implicaron que muchos medicamentos que eran anunciados libremente en la prensa colombiana fueron sometidos a control, lo que disminuyó la aparición de publicidad dirigida a la madre. Esto trajo consigo una renovación de productos y, por lo tanto, de la publicidad que a partir de la década de 1930 estaba mayormente enfocada en la niñez y al papel de la madre en el cuidado de su prole, como veremos a continuación.

## “Niño saludable, madre feliz”: purgantes, vitaminas y pomadas

Si bien entre 1903 y 1929, en la prensa colombiana, se anunciaban medicamentos para la niñez, siendo estos principalmente los vermífugos (¡Madres!..., 22 jul. 1917, p.3), fue a partir de la década de 1930 que las empresas publicitarias y de medicamentos colocaron el enfoque en estos, ofreciendo una variedad de productos en búsqueda de conservar la salud de los infantes. Esta preocupación por la niñez estuvo ligada a los ideales de modernidad y progreso promulgados por el gobierno liberal que asumió la dirección del Estado entre 1930 y 1946. Así mismo, las mujeres fueron vistas como las encargadas de la educación de los menores y de la formación de los futuros ciudadanos de la nación bajo los preceptos promulgados por los médicos que buscaron a través de la madre actuar sobre la población infantil (Rodríguez, 1998, p.20; Colangelo, 2018).

Por esta razón, la industria farmacéutica empleó en sus avisos la misma estrategia utilizada para dirigirse a las mujeres, pero esta vez utilizándolas a ellas como interlocutoras en la medicalización de la niñez. Este proceso fue puesto en marcha entre 1930 y 1945, donde al igual que sucedió con los fármacos para la madre, recurrieron a las medidas de autocuidado que ejercían las mujeres sobre su familia para introducir una variedad de específicos. En la publicidad difundida en la prensa colombiana, este discurso empezó a hacerse mucho más evidente a finales de la década de 1920, tomando fuerza en las décadas de 1930 y 1940.

**Figura 3:**
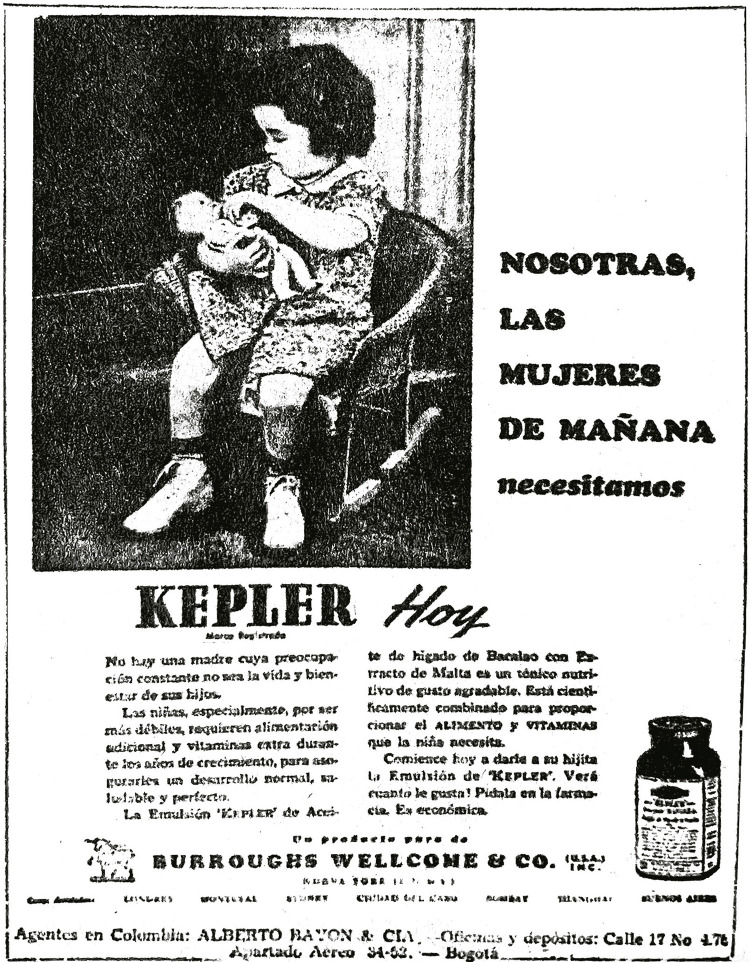
: Nosotras, las mujeres de mañana necesitamos Kepler hoy (Nosotras…, 17 nov. 1944, p.7)

Debido a esto y para captar su atención, le adjudicaron la responsabilidad de la salud y felicidad de su criatura, creando una constante preocupación por el bienestar de estos. Por eso, en la prensa se ratificaba que “no hay una madre cuya preocupación constante no sea la vida y bienestar de sus hijos” (Nosotras…, 17 nov. 1944, p.7). En la imagen reproducida en el siguiente aviso observamos una pequeña niña que tiene en sus brazos una muñeca replicando la figura de la madre que cuida a su bebé, como apoyo a la idea del título del anuncio: “Nosotras, las mujeres de mañana necesitamos Kepler hoy”. A su vez, el objetivo era relacionar lo femenino con lo materno (ver Figura 3).

Así mismo, los avisos adoptaron la idea de brindar supuestos consejos a las madres sobre el cuidado infantil, de este modo empezaron a construir una serie de adjetivos y calificativos para denominar a las madres, entre los que estaban: “prudentes” (15 años…, 1 jul. 1919, p.3) “previsoras” (Metholatum, 22 ago. 1931, p.7), “cuidadosa” (¿Esta el niño…, 21 jun. 1933, p.9) “buena madre” (Madres que…, 28 sept. 1936, p.4) “sacrificada” (Siempre una…, 26 maio 1931, p.4), con el objetivo de construir un modelo ideal materno que a través del acceso y compra de los citados medicamentos lograba garantizar el bienestar infantil. En otras palabras, estas frases buscaban la idea de una mujer sumisa que estaba atenta a los preceptos que la industria farmacéutica le imponían. Un ejemplo de ello es una publicidad de 1936 de J.G.B.,^3^ empresa de medicamentos colombiana, donde se reproducía al final del anuncio una certificación que concluía: “Con mucho gusto y poseída del agradecimiento que como buena madre guardo, recomiendo este producto que da las garantías que buscan en un buen vermífugo” (Madres que…, 4 sept. 1936, p.4). Utilizando las mismas estrategias publicitarias de las empresas extranjeras, para ratificar que una “buena madre” era la que accedía a este producto.

Además, una de las formas de apelar a la capacidad de autocuidado y ejercer procesos de automedicación era lograr que las madres pudieran identificar las enfermedades que aquejaban a sus hijos e hijas. Para ello, utilizaban la estrategia de preguntas y respuestas, verbigracia de esto: “Madres ¿Su hijo está pálido, decaído, le falta apetito?” (Madres…, 30 oct. 1943, p.6); “Señora, ¿Desea saber cuántas lombrices tiene su nene?” (Señora…, oct. 1936, p.6); “¿Está el niño enfermo?” (Esta el niño…, 21 jun. 1933, p.9) y “¿Tose el nene de noche?” (¿Tose el…, 30 ene. 1945, p.2). Estos interrogantes estaban acompañados por respuestas que enumeraban una variedad de síntomas para finalizar introduciendo el medicamento que daba solución al problema presentado.

Otros productos farmacéuticos dirigidos a la niñez eran los multivitamínicos, destacándose la Emulsión de Scott de la firma neoyorquina Scott & Bowne, el cual vendía aceite de hígado de bacalao en “su versión mejorada” (Niño, 2015, p.19). Este específico, uno de los más longevos en el mercado colombiano, empezó a publicitarse a finales del siglo XIX (p.15). Sus avisos casi siempre venían acompañados de un pescador que cargaba un bacalao en su espalda. Fue uno de los primeros medicamentos en incluir la imagen como un elemento clave en su publicidad (Arango, 2007, p, p.116).

Si bien, la Emulsión de Scott publicaba una variedad de avisos que iban dirigidos al público en general, su estrategia publicitaria estaba enfocada principalmente en las madres y sus descendientes, presentándose como una importante ayuda en el proceso de crecimiento y desarrollo infantil. Al igual que otros medicamentos informaba: “¡Niño saludable, madre feliz!” (¡Niño saludable…, 14 abr. 1942, p.4) relacionando el bienestar de ella con el estado de salud de su prole, lo que, según los publicistas, proporcionaba “una valiosa ayuda para la futura madre (Valiosa ayuda…, 16 dic. 1943, p.11).

La Emulsión de Scott tomó el discurso científico, presentándose como una solución a una variedad de enfermedades, ofreciéndose para tratar desde resfriados comunes, mejorar el sueño del infante, ayudar con la dentición y el raquitismo (Désela…, 11 dic. 1943, p.11). Pero, lo que más se subrayaba era que este multivitamínico ayudaba al crecimiento normal de los niños y niñas, anunciando que “cada día su nene ‘tiene’ que adelantar. Ayúdelo para un crecimiento sano y robusto, dele Emulsión de Scott. Cuanto antes, mejor. Enriquece la sangre, fortifica el organismo entero” (Encamínelo…, 6 oct. 1932, p.7; destacados en el original).

Pero, Emulsión de Scott no era el único multivitamínico encontrado bajo la gama de los aceites de hígado de bacalao, disputándose el nicho del mercado colombiano. La prensa, anunciaban con igual intensidad la Vitaemulsión Uribe Ángel (Vitaemulsión Uribe…, 30 ene. 1937, p.19), producto de fabricación nacional, la Ozomulsión (Sus niños…, 14 maio 1932, p.4), la preparación de Wampole (Una mujer…, 4 sept. 1929, p.4) y el Morrhuol Chapoteaut (Morrhuol…, 22 ago. 1903, p.1). Todos estos usaban las mismas estrategias, al presentarse como una solución a los problemas y enfermedades de los niños y mujeres. Prometían “criar niños sanos” (Vitamulsión, 11 sept. 1936, p.6), presentándose como “el amigo de las madres” (Dele…, 18 jul. 1932, p.4) y utilizaban la publicidad para presentar supuestos “consejo a las madres” (Consejo…, 23 maio 1931, p.4) que introducían el consumo del medicamento publicitado.

A causa de esta competencia, Emulsión de Scott empezó a sugerir a las madres “rechazar sustitutos”, incluso cuestionaban el tipo de medicamento que adquirían. Producto de esto, en los anuncios exageraban la escritura con el uso excesivo de mayúsculas y signos de admiración para enfatizar y hacer más llamativo el mensaje, como lo veremos a continuación:

¿No necesitan la medicina MEJOR? Deles EMULSIÓN de SCOTT no imitaciones, ni sustitutos. ¡Ni aceites sin emulsionar! ¡No pueden ser tan buenos como Scott! Porque en la Emulsión de Scott el aceite de bacalao es elaborado fresco y es refinado por proceso tan especial, que es de 4 a 5 veces más digerible... Y así Scott se transforma en vitalidad... resistencia... Dé, pues, SCOTT a sus niños. ¡Y tómela usted misma! Ningún aceite, emulsión o pastilla tiene las mismas propiedades de Scott (¡Para sus…, 14 oct. 1936, p.9).

De igual manera, los otros productos también empezaron a reseñar que ellos eran el legítimo aceite de bacalao, y en la publicidad señalaban que escogieran el que tuviera la contramarca, “Lúa” (Vitamulsión, 11 sept. 1936, p.6). Así mismo, se afirmaba que productos como Ozomulsión “era fácil de asimilar y 1200 veces mejor que la leche” (Dele…, 18 jul. 1932, p.4). Lo que vemos aquí es cómo a través de la publicidad se generó un campo de disputa sobre las madres y las decisiones que estas tomaban sobre su descendencia, creando tensiones entre las marcas de medicamentos para hacerse con este mercado y a través de ello lograr incidir en la maternidad y en la posibilidad que ellas tenían de medicar y automedicarse.

Otra gama de específicos eran los que se enfocaban en la etapa de la dentición, señalada como una fase que venía acompañado de malestares y dolores. Algunos de estos se presentaban como una ayuda al proceso de formación y aparición de los dientes, entre estos tenemos a la ya mencionada Emulsión de Scott (Encamínelo…, 6 oct. 1932, p.7), el Aceite Vigantol (Tengo…, 11 ago. 1932, p.4) y Valtine (Consejo a las…, 6 jun. 1931, p.4), los cuales eran anunciados como un suplemento de vitamina D. Algunos estaban enfocados exclusivamente en aliviar las molestias que podría traer este proceso, entre esos tenemos el Vino y Jarabe de Dusart, anunciado para evitar que el crecimiento de los dientes fuese “sin cansancio ni convulsiones” (Vino…, 2 sept. 1903, p.1), y el denominado Bromural “Knoll”, recomendado para el “bebé inquieto y llorón. El inofensivo calmante de los nervios” (Bromural…, 19 jun. 1937, p.3).

Un medicamento de patente que era reconocido y contaba con una buena publicidad en la prensa colombiana era el llamado Jarabe Calmante de la Sra. Winslow, patentado en 1835 por la farmacéutica Curtis & Perkins (Arango, 2006, p.114), que prometía mantener al “bebe saludable y contento durante la dentición” (Su niñito…, 8 nov. 1943, p.4) y repetía la estrategia ya usada por la Emulsión de Scott de relacionar la salud del bebé con la felicidad de la madre (Niño saludable…, 14 abr. 1942, p.4). Este medicamento afirmaba que no contenía “narcóticos o alcohol”, respondiendo a los ataques que recibió en su país de origen, donde a principios del siglo XX fue denunciado por contener altas dosis de opiáceos (Cowen, Helfand, 1999, p.180; Denham, 2020, p, p.125).

**Figura 4:**
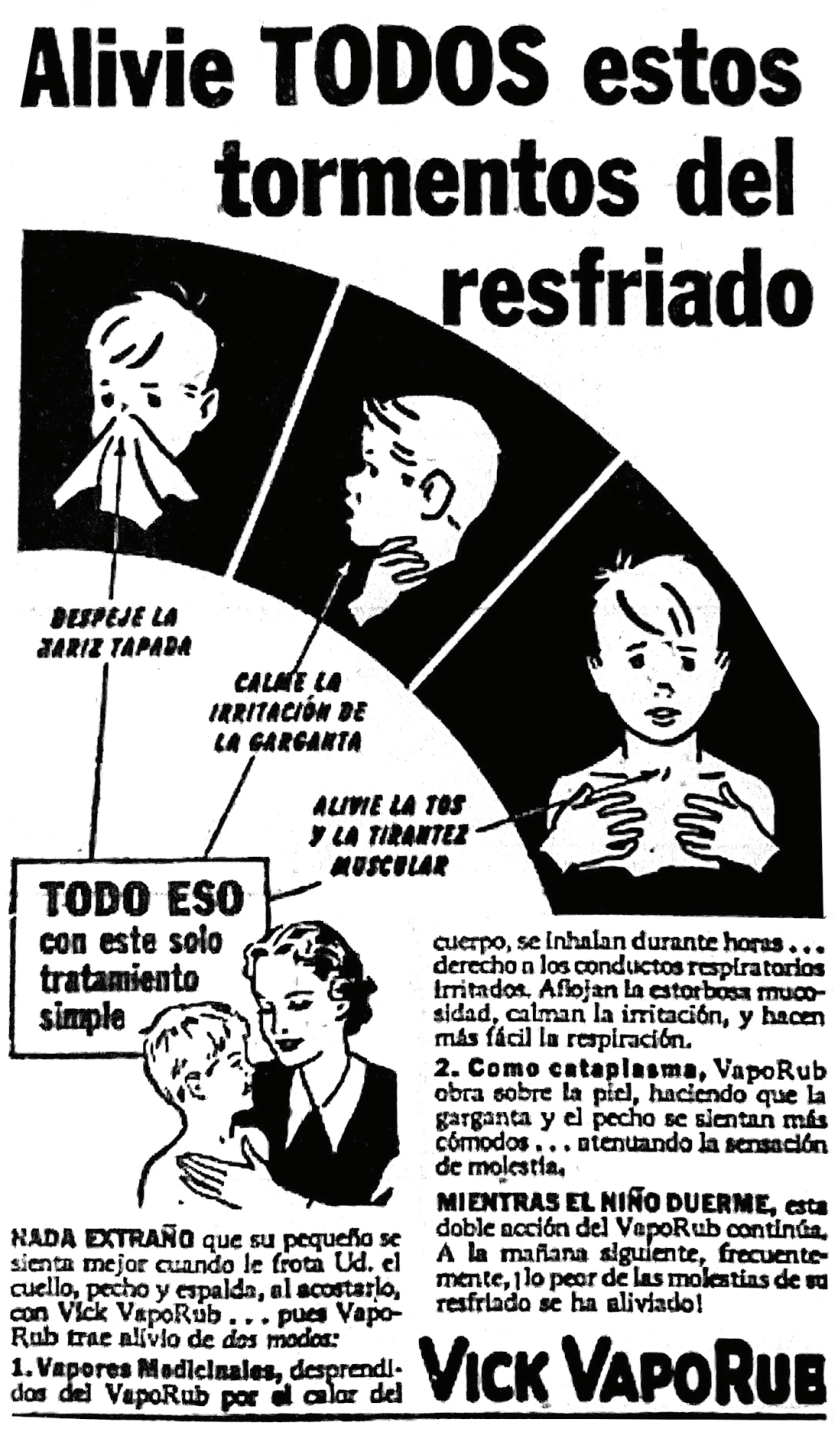
: Alivie todos estos tormentos del resfriado (Alivie…, 9 mar. 1945, p.7)

Los mentolados eran otra gama de medicamentos “ideales para los niños” que eran empleados para el tratamiento de los catarros; uno de los más populares era Vick Vaporub. La publicidad señalaba que “en 61 países, las madres de ideas modernas no obligan más a los niños a tomar medicinas desagradables para los resfriados. En lugar de esto, simplemente les frotan Vick Vaporub en el pecho y cuello” (Ideal…, 7 nov. 1931, p.6). Este mecanismo fue usado por otras publicidades vinculando la idea de que usar este producto era estar inmerso en la modernidad, a la par de sus homólogas extranjeras, utilizando la idea de una maternidad transnacional. Así mismo, mucha de su publicidad iba acompañada de una imagen de los síntomas que se aliviaban con el uso del producto acompañado de una pequeña imagen en donde aparece una amorosa madre frotando el pecho (ver Figura 4).

Otro producto que atendía la misma gama de enfermedades era el Mentholatum, que empleó el prestigio médico y científico para promocionar, mencionando de forma genérica que era recomendado por facultativos y enfermeras (Metholatum, 18 jul. 1931, p.6); mientras que, en otro anuncio, utilizaba la construcción del ideal materno, al decir “las madres previsoras siempre lo tienen a la mano” (Metholatum, 22 ago. 1931, p.7). Además de anunciarlo para los resfriados y catarros, era recomendado para herpes, sarpullidos, irritaciones, comezón del pañal, picaduras de insectos, quemaduras y neuralgias (Metholatum para…, 25 oct. 1932, p.6). Era presentando como una panacea, un solo ungüento para múltiples enfermedades, de ahí que, entre más síntomas o malestares enunciados, mayores posibilidades existía que el público mostrara interés por este y efectuara la compra del mismo.

Todos los específicos aquí estudiados, publicados principalmente entre 1930 y 1945, tomaron como interlocutora a la mujer en su rol de madre con el objetivo de incidir en los procesos de crianza. Las empresas farmacéuticas vieron en ellas un nicho de mercado donde establecer sus productos y así desarrollar una gama de medicamentos que permitieran fortalecer, desparasitar y curar las afecciones infantiles. Estos, como los que iban dirigidos a las mujeres en particular, usaron las mismas estrategias que ayudaron a configurar roles de género.

## Consideraciones finales

A través de los anuncios publicitarios se construyó un discurso que reflejó una serie de estereotipos en torno a la maternidad, trayendo consigo un producto farmacéutico para cada problema. Esto tuvo dos efectos, el primero de ellos fue que al replicar este tipo mensajes la publicidad de los medicamentos reforzaba los imaginarios alrededor de la maternidad, y segundo, al crearse un remedio para cada etapa, trajo consigo un proceso de medicalización al convertir en una cuestión patológica cada aspecto de la procreación y la crianza.

Es así, como, a través del comercio de medicamentos para la madre, se puede comprender la medicalización de la maternidad y la crianza generando un proceso dinámico que trajo consigo la configuración de un ideal materno. Este proceso era un paso necesario para que los medicamentos pudieran encontrar un nicho de mercado lo suficientemente grande para dirigir la publicidad a esta población. Para lograrlo, la industria farmacéutica se mezcló con el género y se apoyó en los discursos científicos vigentes para trasformar los modos de ejercicio de la maternidad, ayudando a construir un prototipo de madre cuyas características principales era saber leer y escribir, estar dedicada a su hogar y a la crianza de su prole. Esta figura solo era posible en las capas medias y altas de la sociedad colombiana.

Pero, los medicamentos no solo eran dirigidos a la experiencia materna, sino que a partir de la década de 1930 buscaron incidir en los modos de crianza, generando una publicidad para cada etapa o problema infantil. Todo esto en búsqueda de posicionarse en el mercado colombiano con una oferta fuerte de productos farmacéuticos. Bajo estas premisas se usaron estereotipos de género que buscaron mostrar que una buena madre siempre estaba feliz y sus hijos saludables. Si bien, no es posible evaluar el impacto que tuvo esta publicidad directamente sobre las madres colombianas, sí podemos resaltar que los medios de comunicación fueron un educador informal que incidió en esta experiencia tratando de intervenir y ajustarla a los objetivos del mercado.

Esto provocó que dentro de la publicidad se generara una tensión constante con la figura materna y su prole como nicho de mercado, proceso que se desarrolló de forma paralela a la medicalización que afectaban a ambos. La disputa por este nicho de mercado no solo era ejercida por los anunciantes de productos, sino que a partir de 1930 fue protagonizada por lo médicos que vieron en esto otra forma de control y disciplina de las mujeres colombianas a los preceptos de la maternidad científica.

Por último, en los anuncios aquí estudiados primó la escritura, razón por lo cual la figura textual, las onomatopeyas y los formatos de textos, jugaron un papel importante al momento de realizar los anuncios. Estos elementos van cambiando con el paso del tiempo, cediendo espacio al uso de la imagen como forma central de comunicación en la publicidad, lo que agregó otros elementos de análisis que no fueron parte de este escrito.

## Data Availability

No se encuentran en un repositorio.

## NOTAS

1 El anuncio era el siguiente: “Para las damas, todas las mujeres bellas usan la crema Simón. Mda. Adelina Patti dice: ‘La he encontrado muy buena’ Savon á la creme Simon. J. Simon Pouder Simon Parfums Violette-Heliotrope París, Farmacias, Peluquerías, Perfumerías y Tiendas” (To ladies, 9 dic. 1904, p.4).

2 El título aparece originalmente en negritas y mayúsculas.

3 Empresa colombiana que para la década de 1930 introdujo al mercado colombiano la fabricación de sus propios medicamentos (Obando, 2017, p.157-158).
